# Randomized controlled trial to evaluate the effects of combined progressive exercise on metabolic syndrome in breast cancer survivors: rationale, design, and methods

**DOI:** 10.1186/1471-2407-14-238

**Published:** 2014-04-03

**Authors:** Christina M Dieli-Conwright, Joanne E Mortimer, E Todd Schroeder, Kerry Courneya, Wendy Demark-Wahnefried, Thomas A Buchanan, Debu Tripathy, Leslie Bernstein

**Affiliations:** 1 Division of Biokinesiology and Physical Therapy, University of Southern California, 1540 E. Alcazar St. CHP 155, 90089 Los Angeles, CA, USA; 2 Division of Medical Oncology and Experimental Therapeutics, City of Hope National Medical Center, 1500 E. Duarte Rd, 91010 Duarte, CA, USA; 3 E-488 Van Vliet Center Faculty of Physical Education and Recreation, University of Alberta, Edmonton, Alberta T6G 2H9, Canada; 4 Department of Nutrition Sciences, University of Alabama Birmingham, 1675 University Blvd, 35294 Birmingham, AL, USA; 5 Division of Endocrinology and Diabetes, Keck School of Medicine, University of Southern California, 2250 Alcazar St. Suite 200, 90033 Los Angeles, CA, USA; 6 Department of Medicine, Keck School of Medicine, University of Southern California, 1441 Eastlake Ave, 90033 Los Angeles, CA, USA; 7 Division of Cancer Etiology, Beckman Research Institute, City of Hope, 1500 E. Duarte Rd, 91010 Duarte, CA, USA

**Keywords:** Exercise, Breast cancer, Metabolic syndrome

## Abstract

**Background:**

Metabolic syndrome (MetS) is increasingly present in breast cancer survivors, possibly worsened by cancer-related treatments, such as chemotherapy. MetS greatly increases risk of cardiovascular disease and diabetes, co-morbidities that could impair the survivorship experience, and possibly lead to cancer recurrence. Exercise has been shown to positively influence quality of life (QOL), physical function, muscular strength and endurance, reduce fatigue, and improve emotional well-being; however, the impact on MetS components (visceral adiposity, hyperglycemia, low serum high-density lipoprotein cholesterol, hypertriglyceridemia, and hypertension) remains largely unknown. In this trial, we aim to assess the effects of combined (aerobic and resistance) exercise on components of MetS, as well as on physical fitness and QOL, in breast cancer survivors soon after completing cancer-related treatments.

**Methods/Design:**

This study is a prospective randomized controlled trial (RCT) investigating the effects of a 16-week supervised progressive aerobic and resistance exercise training intervention on MetS in 100 breast cancer survivors. Main inclusion criteria are histologically-confirmed breast cancer stage I-III, completion of chemotherapy and/or radiation within 6 months prior to initiation of the study, sedentary, and free from musculoskeletal disorders. The primary endpoint is MetS; secondary endpoints include: muscle strength, shoulder function, cardiorespiratory fitness, body composition, bone mineral density, and QOL. Participants randomized to the Exercise group participate in 3 supervised weekly exercise sessions for 16 weeks. Participants randomized to the Control group are offered the same intervention after the 16-week period of observation.

**Discussion:**

This is the one of few RCTs examining the effects of exercise on MetS in breast cancer survivors. Results will contribute a better understanding of metabolic disease-related effects of resistance and aerobic exercise training and inform intervention programs that will optimally improve physiological and psychosocial health during cancer survivorship, and that are ultimately aimed at improving prognosis.

**Trial registration:**

NCT01140282; Registration: June 10, 2010

## Background

Recent estimates show that within the U.S. there are more than 2.4 million breast cancer survivors with a 5-year survival rate of 88.6% [1]. These survivors are at an increased risk of cancer recurrence, comorbidities such as diabetes, osteoporosis and cardiovascular disease, and premature death [2,3]. They also have special needs as a consequence of the adverse effects associated with common treatments, such as surgery, chemotherapy, radiation therapy, and endocrine therapy. One important consequence of these adverse effects is a profound decline in physical activity [4]. This is of particular concern as physical activity is thought to lower the risk of cancer recurrence and mortality [5]. Studies of survivors 6–12 months following treatment describe individuals with decreased cardiorespiratory function, muscle strength, bone mineral density, and physical well-being [6,7]. In addition, these survivors experience fatigue, depression, anxiety, and weight gain [8-10]. These effects of decreased physical activity appear to have more profound effects on cardiovascular and psychosocial health in those undergoing radiation and some forms of chemotherapy [3,11]. While the effects between decreased physical activity and factors of overall well-being has been established in moderate and long-term survivors [12-15], less information is available with regards to ‘early’ (0–6 months post-treatment) survivors.

In 2012, the American Cancer Society released guidelines for cancer patients and survivors promoting physical activity to improve cancer outcomes [16], emphasizing the beneficial effects of exercise for the health of breast cancer survivors. Not only has physical activity been associated with decreased breast cancer risk [17-19], it has also been shown to be beneficial for breast cancer survivors. For example, aerobic exercise has been found to improve cardiorespiratory function in survivors 6 months to 5 years post-treatment [12,13,20,21], which is particularly important for survivors who have had cardiac damage from chemotherapy. Resistance exercise has been found to increase muscle strength [7,14,22,23], which subsequently reduces injuries and improves balance. Additionally, combined (aerobic and resistance) exercise programs result in improvements in cardiorespiratory function and muscle strength in breast cancer survivors [24-26]. Combined exercise involves both resistance and aerobic exercises in a single session and therefore, is effective in improving cardiovascular, musculoskeletal, and psychological health.

Current evidence suggests that breast cancer treatments, such as chemotherapy, lead to weight gain and increased adiposity, fatigue, physical inactivity, and negative alterations in components of metabolic syndrome (MetS) [4,10,27]. Metabolic syndrome (MetS), which is associated with increased risk of cardiovascular diseases and type 2 diabetes (6), is a cluster of risk factors including visceral adiposity, hyperglycemia, low serum high-density lipoprotein cholesterol, hypertriglyceridemia, and hypertension [28]. MetS is highly prevalent and present in at least 25% of American and European adults [29]. Therefore, despite high breast cancer survival rates, many breast cancer survivors are at risk of and may experience mortality from diabetes and cardiovascular disease, which can be modified by lifestyle interventions [30,31]. Obese postmenopausal breast cancer survivors receiving adjuvant hormone therapy present with MetS and elevated levels of C-reactive protein, placing them at a higher risk for cardiovascular and metabolic diseases [27,32,33]. Premenopausal breast cancer patients experience detrimental effects such as increased body mass index (BMI) and central obesity from adjuvant chemotherapy potentially contributing to MetS [34]. Chemotherapy in premenopausal breast cancer patients frequently induces premature menopause, which is associated with increases in body fat, cholesterol, and triglycerides [34,35]. These changes may contribute to earlier development of cardiovascular disease or type 2 diabetes among women already at risk or to increased risk among those not already predisposed to these diseases [28].

Treatment and management of MetS mainly consists of symptomatic drug treatments of the syndrome’s individual components [36]. Candidate drugs that reduce hyperglycemia may have additional metabolic benefits. Examples are metformin, currently being evaluated in a large randomized adjuvant trial for early stage breast cancer [37], PPARγ agonists, GLP-1 agonists, and DPP-4 inhibitors [36,38]. However, since lifestyle factors such as physical activity, dietary intake, and smoking habits affect the risk of developing MetS, it is important and possibly preferable to target lifestyle factors to prevent the onset of metabolic-related diseases in breast cancer survivors. The few studies that have examined the effects of exercise training on particular components of MetS in postmenopausal breast cancer survivors have shown reduced insulin levels and waist circumference, but no change in insulin resistance, fasting glucose, or body weight [39,40]. Some of these results are promising and emphasize the need for additional studies in this area. Studies of premenopausal survivors will be particularly important. Given that chemotherapy induces many of the components of MetS, an effort to offset potential treatment side effects can greatly benefit breast cancer patients.

This trial is a prospective, randomized controlled trial (RCT) in breast cancer survivors following chemotherapy and/or radiation treatments exploring the effects of a 16-week supervised progressive combined (aerobic and resistance) exercise intervention on components of MetS (visceral adiposity, glucose, high-density lipoprotein cholesterol, triglycerides, and blood pressure), as well as muscular strength, cardiorespiratory fitness, and body composition.

### Objectives

The primary objective of this trial is to determine the effects of a 16-week combined exercise intervention on components of metabolic syndrome (MetS) including waist circumference, blood pressure, fasting glucose, HDL, triglycerides, and glucose, compared to a usual care control group among breast cancer survivors. Secondary objectives are to determine the effects of exercise on cardiorespiratory fitness, muscle strength, body composition, quality of life, shoulder strength and range of motion (ROM), and serum levels of insulin, C-reactive protein, and glycosylated hemoglobin (HbA1c). Safety and feasibility of the supervised exercise program will be evaluated, and the sustainability of the effects will be assessed.

## Methods/Design

### Study design

**Figure 1:**
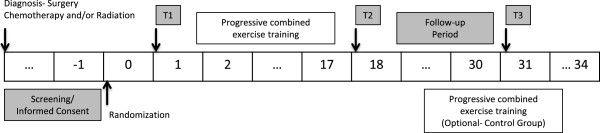
**Study design.** Endpoints are assessed at baseline (T1), after the end of the 16-week intervention (T2), and 12 weeks post-intervention (Exercise Group only; T3).

**Figure 2:**
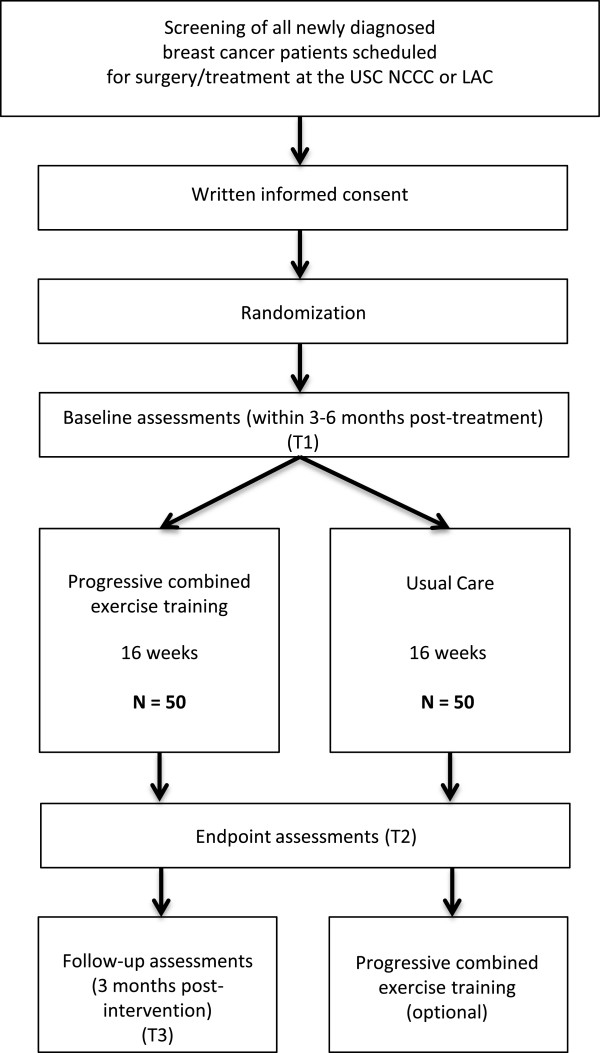
Study flow.

This RCT targets women with stage I-III breast cancer following radiation and/or chemotherapy treatments. After obtaining written consent, participants are randomized to a supervised combined (aerobic and resistance) exercise program over 16 weeks (Exercise Group) and usual care (Control Group; see Figure 1). Endpoints are assessed at baseline (T1), after the end of the 16-week intervention (T2), and 12 weeks post-intervention (Exercise Group only; T3; see Figure 2).

To enhance participation rate and maintain high compliance to the intervention scheme, participants in the Control group are offered the exercise program following the study period. Participants in the Exercise group are supervised by a certified exercise specialist to ensure a tailored, personalized program is executed. The exercise intervention is based on a previously utilized program deemed safe and effective in breast cancer survivors [40].

This study was approved by the Institutional Review Board of the University of Southern California (HS-12-00141) and is registered at ClinicalTrials.gov (NCT01140282).

### Participant selection

This trial includes women with histologically confirmed Stage I-III primary breast cancer who have recently completed all cancer-related treatments and who do not have any contraindications for moderate exercise. Inclusion and exclusion criteria are provided in Table 1.

**Table 1:** Inclusion and exclusion criteria

**Inclusion criteria**	**Exclusion criteria**
• Women with primary breast cancer, stage I-III	• History of chronic disease (i.e., diabetes, uncontrolled hypertension, thyroid disease)
• ≥ 18 years of age	• Weight reduction > 10% within past 6 months
• Have undergone lumpectomy or mastectomy	• Metastatic disease
• Have completed neoadjuvant/adjuvant chemotherapy and/or radiation therapy	• Planned reconstructive surgery with flap repair during trial or follow-up period
• Initiate exercise program (if randomized to that arm) within 24 weeks of therapy completion	• Cardiovascular, respiratory, or musculoskeletal or joint problems that preclude moderate physical activity
• BMI ≥ 25 kg/m^2^ or body fat > 30%	
• Currently participates in < 60 minutes of physical activity per week	
• May use adjuvant endocrine therapy if use will be continued for duration of study period	
• Nonsmokers (No smoking during previous 12 months)	
• Willing to travel to USC	
Able to provide physician clearance to participate in exercise program	
• Women of all racial and ethnic backgrounds will be included in the study enrollment process	

### Recruitment and randomization

All eligible breast cancer patients scheduled for chemotherapy and/or radiation at the USC Norris Comprehensive Cancer Center (NCCC) or Los Angeles County Hospital are briefly informed about the trial during their medical oncology or surgical oncology appointments. If interested, patients are then informed in detail about the trial by the study principal investigator and inclusion/exclusion criteria are verified. For each patient recruited into the study, written informed consent is obtained prior to performing randomization or outcome measure testing. Upon written informed consent, the patient is randomly assigned to either the Exercise or Control groups, and scheduled for the baseline visit, which should be within 0–24 weeks of completing chemotherapy and/or radiation. Randomization (intervention assignment) is done by the Clinical Investigation Support Office (CISO) at the USC NCCC; study investigators contact CISO once the participant has signed the informed consent and is registered. To randomly assign the intervention arm to patients, 2 randomization lists are prepared in advanced to accommodate the stratification: one for pre-menopausal participants and one for post-menopausal participants, based on their menopausal status at time of cancer diagnosis. The randomization is based on a permuted block design with block size of 10. Stratification is used in the randomization process, as we expect menopausal status to have a major influence on our study outcomes. To prevent possible bias, study personnel involved in the recruitment do not have access to the randomization lists. Conversely, the biostatistician does not have influence on the recruitment procedure.

### Exercise intervention

Participants in the Exercise group participate in the exercise program three times per week for 16 weeks for approximately 60 minutes per session, totaling 48 sessions. Participants train one-on-one with a Certified American College of Sports Medicine/American Cancer Society (ACSM/ACS) Cancer Exercise Trainer for each session. Aerobic exercise duration per session is increased by 5 minutes every 3-4 weeks resulting in a total of 45 minutes of aerobic exercise per session by week 16 of the intervention. The trainer documents attendance of each participant at each session. If sessions are missed, reasons are documented with make-up sessions allowable extending the program to a maximum of 18 weeks.

Sessions are comprised of machine- and free weight-based resistance exercise and aerobic exercise at the Clinical Exercise Research Center (CERC) in the Division of Biokinesiology and Physical Therapy at USC. Estimated one-repetition maximum (1-RM) and estimated VO_2_ maximum obtained during baseline testing is used to determine resistance load for each exercise and aerobic intensity, respectively. The exercise program (Table 2) complies with American College of Sports Medicine (ACSM) exercise guidelines for cancer survivors, which includes 20–60 minutes of aerobic exercise performed at least 3 times per week and 6–10 resistance exercises performed at least 1–3 days per week [41-43]. Participants are required to wear a Polar® heart monitor (Lake Success, NY) during each exercise session. Heart rate (HR) is monitored throughout the aerobic exercise sessions to maintain an exercise HR at 65-80% of maximum HR. This protocol is designed to include moderate-to-vigorous forms of activity, which are more beneficial in decreasing risk of mortality from breast cancer, cardiovascular disease and diabetes [30,31,44]. Aerobic exercise types include treadmill walking, jogging, hill walking, or stationary cycling. Each resistance exercise session includes the following exercises: 1) leg press; 2) lunges; 3) leg flexion; 4) leg extension; 5) chest press; 6) seated row; 7) biceps curls; and 8) triceps pushdown. Initial resistance is set at 80% of the estimated 1-repetition maximum (1-RM) for lower body exercises and 60% 1-RM for upper body exercises. When the participant is able to complete 3 sets of 10 repetitions at the set weight in 2 consecutive sessions then the weight is increased by 10%. Participants with a compression garment were required to wear the garment during the exercise sessions. Each daily session begins with a 5 minute warm up on the treadmill or cycle and 10 minutes of static stretching.

**Table 2:** Combined exercise intervention

**Day 1**	**Day 2**	**Day 3**	**Day 4**	**Day 5**	**Day 6**	**Day 7**
Aerobic exercise: 30 minutes at 65-80% HR maximum; Resistance exercise: 3 sets of 10 repetitions, 45 second rest between sets	Rest	Aerobic exercise: 30 minutes at 65-80% HR maximum; No resistance exercise	Rest	Aerobic exercise: 30 minutes at 65-80% HR maximum; Resistance exercise: 3 sets of 10 repetitions, 45 second rest between sets	Rest	At-home aerobic exercise: 30 minutes at 65-80% HR maximum

### Follow-up period

After the 16-week intervention period has ended, participants in the Exercise group are asked to return to the CERC 12 weeks later to repeat the outcome measure testing. During the 12-week period, participants are encouraged to exercise on their own without study team supervision. This can be independent or group activity and participants may seek assistance from fitness professionals as they see fit. The participants are asked to maintain weekly physical activity logs and wear a pedometer on a daily basis during this period. The purpose of this follow-up period is to determine whether the participants remain active and can maintain the benefits gained from the exercise intervention.

### Outcome measures

The outcome measures tested in the study are summarized in Table 3.

**Table 3:** Assessments and instruments used

**Outcomes**	**Instrument**	**T1**	**T2**	**T3**
**Primary endpoint**				
Components of MetS (5):		X	X	X
Biomarkers- Glucose, HDL, triglycerides	Analyzed in peripheral blood	X	X	X
Blood pressure	Pressure cuff under resting conditions	X	X	X
Waist circumference	Measuring tape	X	X	X
**Secondary endpoints**				
Cardiorespiratory fitness	4-minute walk test	X	X	X
Muscle strength	10-RM- leg extension, leg flexion, chest press, seated row	X	X	X
Body composition	DEXA, weight, height, lean mass, % body fat, hip circumference	X	X	X
Quality of life	SF-36, FACT-B, CES-D	X	X	X
Shoulder strength	Muscle force for SE and ER	X	X	X
Shoulder ROM	Measured with goniometer at 90° ER, forward flexion,	X	X	X
Upper limb musculoskeletal disorder assessment	DASH, PSS	X	X	X
Biomarkers- inflammation & endocrine function	Analyzed in peripheral blood	X	X	X
**Others**				
Breast cancer characteristics	Family history, TNM status, ER/PR status, HER-2 score	X		
Medical history	Recording of any pre-existing disease and allergies	X		
Treatment data	Pre-treatment: date and type of breast cancer, affected lymph nodes, chemotherapy type and dose, radiation- technique, type, dose, start/stop date, hormone therapy	X		

### Metabolic syndrome

The primary endpoint is change in the components of metabolic syndrome (MetS) from baseline to week 17. MetS is comprised of 5 components: hypertension, high waist circumference, hyperglycemia, low HDLs, and elevated triglycerides; an individual is diagnosed with MetS if they present with 3 out of the 5 components [45]. Therefore, at each time point, the 5 MetS components are assessed individually and each participant is given a score out of 5 based on the criteria used to determine whether each component lies within the normal range. The following criteria was used to score MetS: 1) blood pressure > 130/85; 2) waist circumference > 35 inches; 3) triglycerides ≥ 150 mg/dL; 4) HDL < 50 mg/dL; 5) glucose ≥ 100 mg/dL. Upon study completion, we will analyze changes in each component as well as the cumulative score.

#### Venipuncture

Fasting blood (~30 cc) is drawn from the antecubital vein by trained phlebotomists at the Clinical Exercise Research Center (CERC). Participants are asked to fast for 12 hours prior to the blood draw. Refreshments are provided following the blood draw and prior to proceeding with further testing.

#### Biomarkers

The Diabetes and Obesity Research Institute (DORI) at the USC performs appropriate standard assays to measure serum lipids (cholesterol, HDL-cholesterol, LDL-cholesterol, triglycerides), insulin, glucose, high sensitivity C-reactive protein (CRP), and glycosylated hemoglobin (HbA1c) from the peripheral blood samples. Fasting glucose, insulin, and HbA1c serve as metabolic biomarkers while CRP serves as an inflammatory biomarker [27]. Serum is derived from whole peripheral blood samples, processed within 2 hours after taking the blood sample and stored at −80° for analyses of biomarkers after completion of last study participant.

#### Waist circumference

A tape measure is used to obtain waist circumference measured at the midpoint between the lower margin of the last palpable rib and the top of the iliac crest. Hip circumference is measured, around the widest portion of the buttocks, with the tape parallel to the floor, to determine waist-hip ratio.

#### Blood pressure

The participant is asked to sit quietly for 5 minutes while resting her arm (ipsilateral to the affected breast) on a table so the brachial artery is level with the heart. A sphygmomanometer cuff is wrapped around the participant’s upper arm, just above the elbow and a stethoscope is placed on the hollow of the elbow so blood pressure can be measured.

### Physical fitness

All fitness measures are performed by trained study personnel at the CERC.

#### Cardiopulmonary fitness

A single-stage submaximal treadmill test is used to estimate maximal oxygen uptake [46]. Cardiopulmonary fitness testing is well established in cancer patients and recommendations for testing procedures as well as safety guidelines in clinical trials with cancer populations have been well defined [47]. The procedure is also used to exclude exercise-contraindicating cardiac impairments. Participants are instructed to walk so they are able to talk while walking at a speed of 2.0, 3.0, 4.0, or 4.5 mph on a treadmill (Desmo Woodway, Waukesha, WI) for 4 minutes at a 5% grade and heart rate is measured at the end of the test. Using HR, speed, age and gender, maximal oxygen uptake is calculated using the specific formula that applies to this test.

#### Muscle strength

Maximal voluntary strength is evaluated by the 10-repetition maximum (10-RM) method for the following exercises: chest press, latissimus pulldown, knee extension, knee flexion, (Tuff Stuff, Pomona, CA) which will be used to calculate 1-RM (maximum strength) values for the exercise intervention [48]. The study personnel demonstrate each exercise and then the participant is asked to complete 5 repetitions of a light weight to ensure proper form. The participant rests for 30 seconds and then is asked to lift a heavier weight 10 times. This is repeated until the participant is no longer able to complete 10 successful repetitions.

#### Body composition

BMI in kg/m^2^ is calculated from height and weight measurements obtained using a medical scale (Detecto® 437, Webb City, MO). Body composition (total lean mass and percent body fat) is measured from a whole body scan using Dual-Energy X-ray Absorptiometry (DXA, GE iDXA, Fairfield, Connecticut) and with bioelectrical impedance analysis (BIA, Biospace InBody 520, Cerritos, CA). The DXA is considered to be a valid and reliable reference method for body composition assessment [49]. BIA is a quick and non-invasive method, which determines electrical impedance, or opposition to the flow of an electric current through body tissues to calculate an estimate of total body water, fat-free mass and body fat [50]. We chose to include the DXA and BIA to assess ability to use more portable techniques such as BIA for future studies and to better determine possible differences between the two devices in this population.

### Shoulder health and lymphedema measures

#### Shoulder strength

Maximal muscle force produced by the primary agonist during scapular plane elevation (SE) and external rotation (ER of the upper extremity are measured using a hand held dynamometer (HHD; Hoggan Health Industries, Salt Lake City, UT). The participant sits in an armless chair with her back flush to the back of the chair, feet flat on the floor approximately shoulder width apart, and sitting with neutral posture. The participant is asked to push as hard as possible against the HHD for approximately 4 seconds. For each test (SE and ER), the dynamometer on the apparatus is aligned so that the resistance is in exactly the opposite direction of the direction of motion being resisted. Two trials are performed for each muscle test, taken sequentially. The subject is allowed to rest for 30 seconds between the two trials. The average of two trials is used for data analysis. Shoulder strength is regularly assessed in breast cancer survivors as a means to determine effects of surgery and treatment-related side effects [51] and is measured following our exercise intervention.

#### Shoulder active range of motion (AROM)

AROM is measured for shoulder forward flexion and shoulder ER at 90 degrees abduction.

##### ER at 90 degrees of abduction

The participant is placed in supine, trunk stabilized on the table, knees bent so that the feet are placed flat on the table. The participant’s arm is placed in 90 degrees of shoulder abduction, the elbow flexed to 90 degrees, and the wrist in neutral position. The participant’s arm is passively moved into external rotation within their pain-free range of motion 3–5 times to precondition the tissue. Then, the participant is asked to perform active ER where ‘active’ refers to the participant moving their own arm through the ROM independent of tester assistance. Active ER is measured with the Acumar™ digital inclinometer aligned between the olecranon and ulnar styloid process to measure external rotation of the glenohumeral joint. Two trials are performed with approximately 10 seconds between each trial.

##### **Forward flexion**

The participant is placed in a standardized seated position, with their back directly against the back of a straight back chair. Participants are asked to actively elevate their arm as far as they can into flexion. The Acumar™ digital inclinometer is aligned along the mid-humeral shaft with the elbow in extension and shoulder in neutral rotation by asking the subject to point their thumb towards the ceiling. Two trials are performed with approximately 10 seconds between each trial.

#### Lymphedema assessment

Geometric arm volume calculations are performed to assess lymphedema. We are calculating arm volume using circumferential measurements taken at anatomic landmarks and was determined to be a reliable and valid method of limb volume measurement [52]. The participant is seated at a table with their shoulder positioned at approximately 90° of scapular plane flexion with their straight arm resting on a table. Circumferential measurements will be taken with a thin plastic tape measure with a spring that standardizes how tightly the tape is pulled. The anatomic landmarks include the wrist to middle forearm, middle forearm to elbow, elbow to middle upper arm, and middle upper arm to a 65% mark. The 65% mark of the upper arm is 65% of the distance from the elbow (olecranon) to the shoulder tip (acromion). Calculations for limb geometric volume will be calculated using the frustum volume as described by Taylor et al. [52] The interrater reliability for this method has been found to be excellent (>0.98)^23^. A percentage difference between lymphedema limb and the uninvolved limb will be calculated to determine the amount of lymphedema. Lymphedema has been defined in recent literature as a greater than 10% difference in volume calculation for the arm compared to the uninvolved upper extremity [52].

### Participant-reported outcomes

#### Physical activity assessments

Physical activity history is assessed at baseline using an interviewer-administered physical activity questionnaire [53]. Throughout the duration of the study period, weekly 7-day physical activity logs [54] are completed by all participants and returned to the PI by mail for the Control group.

#### Dietary assessments

Dietary history is measured at baseline using the NIH-DHQ food frequency questionnaire [55]. Three-day dietary records are completed at baseline and at the completion of the study period to assess recent dietary patterns.

#### Quality of life assessments

Quality of life (QOL) is assessed using the SF-36 and FACT-B. The SF-36 is a multi-purpose, short-form health survey with 36 items used to assess physical and mental health [56]. The FACT-B (Functional Assessment of Cancer Therapy-Breast) is a breast cancer-specific questionnaire comprised of 44 items to specifically assess quality of life in breast cancer patients [57].

#### Psychosocial assessments

Depressive symptoms are assessed using the 20-item CES-D (Center for Epidemiologic Studies Depression) scale, which was designed to measure current level of depressive symptomatology [58].

#### Musculoskeletal disorders assessment

Upper limb musculoskeletal disorders is assessed using the DASH (Disabilities of the Arm, Shoulder, and Hand) and Penn Shoulder Scale (PSS). DASH is a 30-item questionnaire designed to measure physical function and symptoms of possible musculoskeletal disorders of the upper limb [59]. The PSS is a 100-point shoulder-specific self-report questionnaire consisting of 3 subscales of pain, satisfaction, and function [60].

### Tracking and monitoring of adverse events

Potential adverse events (e.g., lymphedema, pain, muscle soreness, nausea, etc.) are assessed, graded and attributed using CTCAE, v4.3 and recorded by the exercise trainer at each training session by standardized questionnaires throughout the intervention period. Adverse events reported spontaneously by the patient or observed by study nurses or physicians are similarly assessed and recorded.

### Sample size

The primary aim is to compare changes in overall MetS scores from baseline to week 17 between the Exercise and Control groups. Since MetS has not been investigated in breast cancer survivors at the time of project initiation, we chose to calculate sample size using changes in insulin following exercise in breast cancer survivors. This project is designed to detect a treatment effect based on insulin results with 80% statistical power at a 5% level of statistical significance. Ligibel et al. assessed insulin levels before and after a 16-week exercise intervention in breast cancer survivors [40]. Using their results, a sample size of 80 participants would detect a difference in mean insulin levels of 2.6 μU/ml assuming that the common standard deviation is 4.0 μU/ml using a two group t-test with two-sided 5% level of statistical significance. Therefore, to allow for a 20% maximum withdrawal rate, 100 participants will be recruited, of whom 50 will be premenopausal and 50 postmenopausal.

### Data analysis

The main intervention effect will be assessed on the basis of a comparison between exercisers and controls as defined at randomization, regardless of exercise adherence, i.e., according to intent-to-treat principle. A 2 (group) × 2 (time) repeated measures analysis of variance (ANOVA) will be used to examine changes in MetS score and for each individual component of MetS. Stratification by type of therapy (i.e., surgery, chemotherapy, radiation) may result based on therapy regimens completed by enrolled subjects. Similar analyses as for MetS will also be performed for the secondary endpoints. Correlation analyses will be used to examine the relationship between changes of the various measured endpoints. Regression analyses regarding the repeated measurement design (T1, T2, T3) will be applied to investigate the association between exercise intervention, MetS, cardiopulmonary fitness, muscle strength, body composition, and the QOL dimensions. The influence of other potential confounding factors, such as age, clinicopathologic characteristics, and comorbidities will be explored and accounted for in the analysis. In addition, change in physical activity behavior post intervention will be explored for the follow-up time points using descriptive analysis.

## Discussion

The present study will contribute to the growing field of exercise in breast cancer survivors with respect to several innovative aspects being tested: 1) Examining MetS components collectively following exercise; 2) progressive combined exercise training; 3) inclusion of premenopausal women; 4) inclusion of ‘early’ breast cancer survivors; 5) sustainability effects of 16-week exercise intervention. Therefore, the results of this study will help inform future more definitive trials powered for disease-free and overall survival as to what indices at baseline or following an exercise program might be predictive of long-term benefit, and how might future programs be individualized for maximal benefit.

With the improvement of survival rate, the impact of comorbidity on survival and quality of life among breast cancer survivors has become an important topic. MetS is an established risk factor for cardiovascular diseases and mortality [28,61]. Emerging evidence implicates MetS as a long-term risk factor for cancer, but also that certain cancer therapies might increase risk of developing MetS among cancer survivors [62-64]. The concern for MetS and related risk factors among breast cancer survivors has attracted growing attention [63]. Previous studies have shown that high levels of exercise are inversely correlated with the prevalence of MetS in the general population [65-67]. However, whether exercise participation can reduce the risk of MetS among cancer survivors has been less studied. Exercise was found to be inversely associated with development of MetS in childhood cancer survivors [68]. Data among breast cancer survivors are scarce. To our knowledge, only one study specifically evaluated the association between exercise and MetS among breast cancer survivors and found that exercise participation between 6 and 60 months post-diagnosis was inversely associated with the prevalence of MetS for exercise participation ≥ 3.5 hours/week (30 min/day; OR 0.69, 95% CI 0.48-0.98) [69]. Thus, observational evidence supports benefits of exercise to attenuate MetS and our current trial will determine whether a supervised exercise intervention will result in improved MetS components.

To date there are over 157 ongoing or completed clinical trials involving exercise and breast cancer survivors reported on ClinicalTrials.gov, demonstrating the growing importance of the health and quality of life benefits of exercise for breast cancer survivors. Physiologically, to attenuate MetS in non-cancer persons, implementing a strictly aerobic exercise program has been found effective since aerobic exercise can reduce blood pressure, decrease triglycerides, improve insulin sensitivity, and body fat [70-73]. However there is additional evidence to support the use of resistance exercise to improve insulin sensitivity and glucose tolerance [74-76]. Thus, to investigate effects of exercise on components of MetS, we designed a progressive combined exercise intervention, which includes both aerobic and resistance exercises. Due to benefits of resistance exercise on muscular strength, balance, and bone density, it is critical to the health of our participants to include both resistance and aerobic exercise. Although a limited scope of research is available to describe specific exercise needs of these survivors, a recent report addresses this topic in rural breast cancer survivors [77]. This indicated that survivors prefer to receive face-to-face exercise counseling from an exercise specialist anytime before or after treatment. Accommodating this need is achieved in our present study as we are providing face-to-face exercise instruction soon after treatment is completed in an effort to enhance sustainability of the exercise program by demonstrating that expensive equipment and extraneous costs do not have to be associated with an exercise. Significant value is placed on exercise by survivors who may or may not participate in exercise as means of health promotion and support [78] which reiterates the importance of exercise and the need for a feasible exercise program to encourage participation in exercise.

Our trial includes a unique population of breast cancer survivors, regarding age and ethnicity. The few studies that have examined the effects of exercise training on particular components of MetS have involved postmenopausal breast cancer survivors [39,40]. Premenopausal breast cancer survivors are understudied and may be particularly important due to the different hormonal milieu from postmenopausal women. At USC, particularly the Los Angeles County hospital, approximately 50% of all breast cancer cases are premenopausal, increasing the feasibility of included premenopausal women. Statistically, we will stratify by menopausal status in order to determine if the effects of our trial varies among premenopausal and postmenopausal women. USC treats a diverse population of patients and is conveniently located in the East Los Angeles section of Los Angeles County also near the San Gabriel Valley. East Los Angeles and the neighboring San Gabriel Valley, with a population of approximately 2.5 million people, is distinct in that it contains a large contingency of Hispanics (34%), Asian Americans (20%) and African Americans (15%) who vary widely in socioeconomic position [79]. Additionally, LA-USC County Medical Center welcomes patients with Medi-Cal coverage, which is California’s low income Medicaid program. Overall, breast cancer patients who receive care at USC are 30% Latina, 20% Asian/Pacific Islanders, 30% White, 15% African American and 5% other. Thus, we will have the ability to recruit from a diverse patient pool, with currently 90% of our patient population is comprised of Latina survivors.

Timing of an intervention is a key aspect in order to capture a time point following treatment where survivors change their lifestyle behaviors, often referred to as the “teachable moment [80]”. Currently, the optimal time to participate in an exercise intervention following breast cancer diagnosis is unknown. Capitalizing on the “teachable moment” soon after treatments by enrolling in a supervised exercise program will foster a behavioral change that will be sustainable for longer periods of time due to the positive and encouraging environment elicited through exercise. Breast cancer survivors may be more likely than other cancer survivors to harbor psychological stress of a diagnosis for longer periods of time and may have higher levels of interest in interventions [81], stressing the importance of an early intervention, supporting our inclusion of ‘early’ breast cancer survivors. ‘Early’ breast cancer survivors include women who have completed all cancer-related treatments within 0–6 months of study entry. Since many exercise trials have included survivors from 6 months- 10+ years post-treatment [82], we believe it was critical to include survivors who more recently completed treatment to capitalize on the teachable moment.

A unique component of this study is the inclusion of a 12-week follow-up period of the Exercise group after completion of the trial to measure longer-term adherence. Numerous cancer-related exercise interventions have failed to examine whether the benefits of the intervention persist following study completion despite reporting high (>80%) adherence rates [83,84]. This is an important aspect to investigate mainly since the benefits of exercise diminish due to failure of the participants to maintain a regular exercise program following study completion. Although many exercise interventions strive to instill a behavioral change to a more physically active lifestyle, there is a lack of data on post-intervention adherence. This study will determine whether the positive benefits of exercise can be maintained following a 12-week follow-up period where participants will not be actively enrolled in an investigator-led exercise intervention. Critical information in regards to diet and physical activity patterns and effects on MetS will be obtained during this period which will provide novel information for future intervention studies to achieve the goal of long-term adherence.

## Conclusion

In summary, this exercise trial shall contribute to a better understanding of metabolic-related effects of combined aerobic and resistance exercise in breast cancer survivors who have recently completed cancer-related treatments. The ultimate goal is the implementation of an optimized intervention program to reduce metabolic syndrome and prevent cardiovascular disease and diabetes during survivorship.

## Competing interests

The authors declare that they have no competing interests.

## Authors’ contributions

CDC, JM, WDW, KC, and LB conception, design, trial protocol and initiation of the project. CDC conception and supervision of biospecimen collection and analyses; CDC and ETS conception and supervision of training interventions and physical performance diagnostics; CDC study coordinator, performs endpoint assessments; DT and TB study physicians and medical advice; CDC and DT study and data management; CDC drafted and finalized the manuscript. All authors have read and approved the final manuscript.

## Pre-publication history

The pre-publication history for this paper can be accessed here:

http://www.biomedcentral.com/1471-2407/14/238/prepub
